# Horseradish Peroxidase-Encapsulated Hollow Silica Nanospheres for Intracellular Sensing of Reactive Oxygen Species

**DOI:** 10.1186/s11671-018-2527-0

**Published:** 2018-04-24

**Authors:** Hsin-Yi Chen, Si-Han Wu, Chien-Tsu Chen, Yi-Ping Chen, Feng-Peng Chang, Fan-Ching Chien, Chung-Yuan Mou

**Affiliations:** 1 Department of Chemistry, National Taiwan University, No. 1, Sec. 4, Roosevelt Rd., Taipei, 10617 Taiwan; 2 Graduate Institute of Nanomedicine and Medical Engineering, College of Biomedical Engineering, Taipei Medical University, No. 250 Wuxing St., Taipei, 11031 Taiwan; 3 International PhD Program in Biomedical Engineering, College of Biomedical Engineering, Taipei Medical University, No. 250 Wuxing St., Taipei, 11031 Taiwan; 4 Department of Biochemistry, Taipei Medical University, No. 250 Wuxing St., Taipei, 11031 Taiwan; 5 Department of Optics and Photonics, National Central University, No. 300, Zhongda Rd., Taoyuan City, 32001 Taiwan

**Keywords:** Reactive oxygen species, Hollow silica nanospheres, Horseradish peroxidase, Enzyme delivery, Porous materials

## Abstract

Reactive oxygen species (ROS) have crucial roles in cell signaling and homeostasis. Overproduction of ROS can induce oxidative damage to various biomolecules and cellular structures. Therefore, developing an approach capable of monitoring and quantifying ROS in living cells is significant for physiology and clinical diagnoses. Some cell-permeable fluorogenic probes developed are useful for the detection of ROS while in conjunction with horseradish peroxidase (HRP). Their intracellular scenario is however hindered by the membrane-impermeable property of enzymes. Herein, a new approach for intracellular sensing of ROS by using horseradish peroxidase-encapsulated hollow silica nanospheres (designated HRP@HSNs), with satisfactory catalytic activity, cell membrane permeability, and biocompatibility, was prepared via a microemulsion method.

These HRP@HSNs, combined with selective probes or targeting ligands, could be foreseen as ROS-detecting tools in specific organelles or cell types. As such, dihydrorhodamine 123-coupled HRP@HSNs were used for the qualitative and semi-quantitative analysis of physiological H_2_O_2_ levels in activated RAW 264.7 macrophages. We envision that this HSNs encapsulating active enzymes can be conjugated with selective probes and targeting ligands to detect ROS in specific organelles or cell types of interest.

**Electronic supplementary material:**

The online version of this article (10.1186/s11671-018-2527-0) contains supplementary material, which is available to authorized users.

## Background

Reactive oxygen species (ROS) consisting of radical and non-radical molecules, such as superoxide anions, hydrogen peroxide, hydroxyl radical, singlet oxygen, and peroxynitrite, are continuously produced during aerobic metabolism. Cellular ROS are mainly generated from the mitochondrial electron transport chain (mETC) and are normally counterbalanced by enzymatic (such as superoxide dismutases, catalases, and peroxidases) and non-enzymatic (e.g., vitamins A, C, and E; urate; and bilirubin) antioxidant defenses [1]. However, imbalances in ROS production can lead to oxidative stress and subsequent damage to DNA, fatty acids, proteins, and other cellular components, potentially contributing to diabetes [2], cancer [3], and cardiovascular disorders [4], and neurodegenerative disorders [5] such as Alzheimer’s disease and Parkinson’s disease. Direct imaging and ROS quantification in living cells are highly desirable but very challenging.

Advances in fluorescent microscopy [6, 7] have allowed the development of the noninvasive measurement and imaging of ROS evolution at the single-cell level. To detect ROS, most probes are designed to measure changes in the fluorescence intensity or shifts in the emission wavelength (i.e., ratiometric methods) following oxidization of profluorescent aromatic molecules or deprotection of masked compounds to fluorescent products [8]. Specificity to a particular type of ROS is of importance when designing successful probes; for example, boronate oxidation is utilized as a bioorthogonal reaction approach for studying the chemistry of hydrogen peroxide in living systems [9]. To explore the spatio-temporal dynamics of ROS, several boronate-based probes conjugated with a positively charged phosphonium moiety were generated for mitochondrial targeting [10, 11]. However, their potential for in vivo imaging is limited by their instability in biological milieu, low penetration of tissue barriers, and rapid elimination from the body through the urinary system [12–14]. To overcome such problems, some strategies have been developed either by chemically grafting an additional stabilizing structure onto the probe [15] (e.g., a triethylene glycol chain), developing genetically encoded fluorescent protein-based indicators [16], or applying reaction-based bioluminescent reporters [17] or positron emission tomography (PET) probes for the molecular imaging of ROS [18]. Moreover, several comprehensive studies highlighted nano-formulations as an important design consideration and demonstrated that nanoparticle-based probes can provide mechanistic insights and innovative strategies to image ROS in living organisms with high specificity and sensitivity [19–22]. Enzymes with high catalytic activity and distinct substrate selectivity have also been utilized as clinical diagnostic tools for identifying target analytes. However, the lack of lasting stability and the difficulty in permeating through biological membranes of free enzymes have often limited their applications in complex biological milieu. Even though applying electrode is not suitable for intracellular assays or in vivo imaging, considerable efforts have been devoted to developing horseradish peroxidase (HRP)-incorporated biosensors to determine H_2_O_2_ based on electrochemical methods [23, 24].

In this work, enzymatic nanoreactors, composed of HRP encapsulated in 45-nm hollow silica nanospheres, were synthesized by a water-in-oil (w/o) microemulsion route followed by a mild etching process [25]. Previously, we demonstrated that such hollow nanomaterials can maintain stable activity of the encapsulated enzymes and nanocatalysts while protecting against proteolysis and sintering, respectively [26, 27]. In this work, we evaluated their potential uses as intracellular biosensors by studying the enzyme entrapment efficiency, loading capacity, peroxide reactivity and selectivity, cellular uptake, toxicity, and proliferation effects of HRP@HSNs. Using dihydrorhodamine 123 (DHR123) as a substrate, which has been commonly coupled with HRP to detect intracellular hydrogen peroxide production, interactions between HRP@HSNs and various types of ROS in aqueous solutions were investigated by flow cytometry and fluorescence microscopy. Furthermore, it was demonstrated that utilization of HRP@HSNs with DHR123 can simultaneously image and quantify physiological H_2_O_2_ levels in phorbol 12-myristate 13-acetate (PMA)-stimulated RAW264.7 macrophages. Taken together, the enzymatic nanoreactors of HRP@HSNs have the potential for imaging ROS-associated inflammatory cells in vivo and the encapsulated components may be extended to multiple different enzymes [28], nanoparticles [26], and recognition molecules for synergistic applications.

## Methods/Experimental

### Chemicals and Reagents

Decane, *n*-hexanol (98%), ammonium hydroxide (NH_4_OH, 35 wt%), tetraethyl orthosilicate (TEOS, 98%), 3-aminopropyltrimethoxysilane (APTMS, 95%), and fluorescein isothiocyanate (FITC) isomer were purchased from ACROS. Polyoxyethylene (5) isooctylphenyl ether (Igepal CA-520), HRP type VI-A (HRP), 3,3′5,5′-tetramethylbenzidine (TMB), citric acid, dimethyl sulfoxide (DMSO), and rhodamine B isothiocyanate (RITC) were purchased from Sigma-Aldrich. 2-(4-Iodophenyl)-3-(4-nitrophenyl)-5-(2,4-disulfophenyl)-2H-tetrazolium was purchased from Clontech. DHR123 and PMA were purchased from Cayman Chemical. Hydrogen peroxide (H_2_O_2_, 35%) was purchased from SHOWA Chemical Industry. Tert-butyl hydroperoxide solution (70% in H_2_O) was purchased from Aldrich. Iron (II) perchlorate (Fe(ClO_4_)_2_) was purchased from Alfa Aesar. Ultrapure deionized (D.I.) water was generated by a Millipore Milli-Q Plus system. All reagents were used without further purification.

### Synthesis of Hollow Silica Nanospheres (HSNs)

HSNs were synthesized by a reverse microemulsion system accompanied by a selectively etching method as described in our previous studies [25, 29]. Typically, 20 mL of decane as the oil phase, 1.63 mL of CA-520 as a surfactant, and 550 μL of *n*-hexanol as a co-surfactant were mixed as well as magnetically stirred with a 2-cm PTFE-coated stir bar at 650 rpm. After that, 350 μL of D.I. water were added to the mixture at room temperature, generating a water-in-oil (w/o) microemulsion system. Next, 25 μL of APTMS ethanolic solution (200 μL of APTMS in 1.4 mL of absolute ethanol) and 100 μL of TEOS were added with stirring. After stirring for 10 min, 250 μL of aqueous ammonia (35 wt%) was introduced into the system with stirring at 20 °C. After 10 h, 95% ethanol was added to destabilize the microemulsion system and the solid silica nanoparticles (SSNs) were collected by centrifugation at 11,000 rpm for 20 min. To obtain HSNs, the SSNs were suspended in D.I. water with stirring at 40 °C for 40 min. Next, the HSNs were collected by centrifugation at 11,000 rpm for 20 min and washed with 95% ethanol several times. Finally, the HSNs were suspended and kept in 99.5% ethanol.

### Synthesis of Horseradish Peroxidase-Encapsulated Hollow Silica Nanospheres (HRP@HSNs)

HRP@HSNs were synthesized by a method based on our previous studies [27, 28]. Typically, the synthesis is similar to the procedure above, except that 350 μL of D.I. water was replaced by 350 μL of aqueous HRP (90 μL of 10 mg/mL of an HRP solution in 350 μL of D.I. water). After synthesis, HRP@HSNs were kept in D.I. water at 4 °C.

### Synthesis of FITC-HSNs and HRP@FITC-HSNs

HSNs and HRP@HSNs with incorporated green-emitting fluorescein dye (designated FITC-HSNs and HRP@FITC-HSNs) were synthesized similar to the procedure above, except that ethanolic APTMS solution was replaced by an FITC-APTMS one. An ethanolic FITC-APTMS solution was prepared by mixing 10 mg of FITC and 200 μL of APTMS with 1.4 mL of absolute ethanol under dark conditions for 18 h at room temperature.

### HRP Entrapment Efficiency and Loading Capacity of HRP@HSNs

First, a mixture comprised of HRP (6 mg in 500 μL of D.I. water) and RITC (3 mg in 350 μL of DMSO) was stirred under dark conditions for 24 h at 4 °C. After that, the mixture was transferred to a dialysis membrane composed of regenerated cellulose with a molecular weight cutoff of 12~14 kDa. Then, to remove the unreacted RITC, the dialysis bag was dialyzed against 1 L of D.I. water and gently stirred for 3 days. Finally, the RITC-labeled HRP (designated RITC-HRP) was used to synthesize RITC-HRP@HSNs.

To determine the HRP loading capacity, RITC-HRP@HSNs were dissolved in 1 mL of NaOH (1 M) for 1 h, and the amount of the entrapped RITC-HRP was calculated from a calibration curve established by plotting the fluorescence intensity versus the concentration of RITC-HRP. The fluorescence was measured with a Hitachi F-4500 Instrument at an excitation wavelength of 543 nm and an emission wavelength of 550~650 nm. The HRP entrapment efficiency and loading capacity of HRP@HSNs were defined as follows: entrapment efficiency (%) = mass of RITC-HRP in RITC-HRP@HSNs/initial mass of RITC-HRP; and loading capacity = mass of RITC-HRP in HRP-RITC@HSNs/mass of RITC-HRP@HSNs.

### HRP Activity Assay

To detect the activity of the peroxidase enzyme, a chromogenic substrate of TMB was used. TMB can be converted into a colored product when oxidized by HRP using hydrogen peroxide as the oxidizing agent. First, various concentrations of native HRP and HRP@HSN were prepared in phosphate and citrate buffer (pH 5.2). Then, each solution was supplemented with 50 μL of the TMB solution (20 μM in DMSO) and 50 μL of H_2_O_2_ (20 μM in D.I. water). The reaction was monitored by measuring the absorbance at 655 nm using a microplate reader (BioTek Synergy Hybrid Reader). The activity of HRP encapsulated in HSNs was calculated from the calibration curve of native HRP.

### Reactivity Assay of HRP@HSNs to Various ROS

DHR123 (20 μM) alone or mixed with HRP@HSNs (50 μg/mL) was incubated with various types of ROS (100 μM) in 100 μL of DMEM solution (pH 7.4). The fluorescence emission at 530 nm (λex = 488 nm) was monitored every 5 min for the first 120 min. The ROS investigated were obtained as follows: hydrogen peroxide (H_2_O_2_) and tert-butyl hydroperoxide (TBHP) were prepared from commercially available 32 and 70% aqueous solutions, respectively. Superoxide (O_2_^•−^) was generated from 10 mM stock of potassium superoxide (KO_2_) in DMEM. Hydroxyl radicals (•OH) and tert-butoxy radicals (•OtBu) were produced by the reaction of 1 mM Fe(ClO_4_)_2_ with 100 μM H_2_O_2_ or 100 μM TBHP, respectively.

### Cell Culture and Viability Assay

The RAW264.7 mouse macrophage cell line was obtained from ATCC. RAW264.7 cells were maintained in DMEM with 10% FBS, 100 U/mL penicillin, and 100 μg/mL streptomycin (Gibco) at 37 °C in 5% CO_2_ atmosphere. Typically, 2 × 10^5^ RAW264.7 cells per well were seeded in 24-well plates for the viability assays. After 24 h, cells were washed twice with PBS and incubated with different amounts (0, 50, 100, and 200 μg/mL) of a nanoparticle suspension in serum-free DMEM for 2 h. For the cytotoxicity assay, nanoparticle-treated cells were washed twice with culture medium followed by incubation with WST-1 reagent (Clontech) at 37 °C for 2 h. For the proliferation assay, cells after treatment with nanoparticles for 2 h were allowed to grow in regular growth medium for 24 h followed by incubation with the WST-1 reagent. Cell viability was determined by the formazan dye generated by live cells, and the absorbance at 450 nm was measured, with a reference wavelength of 650 nm, using a microplate reader (Bio-Rad, model 680).

### Cell Uptake Analysis

RAW264.7 cells at 1 × 10^6^ per well were seeded in six-well plates overnight. Then, RAW264.7 macrophages were treated with different amounts (0, 50, 100, and 200 μg/mL) of a nanoparticle suspension in serum-free DMEM media for 2 h. After that, cells were washed three times with PBS and detached by a trypsin-EDTA solution. The uptake of nanoparticles by RAW264.7 macrophages was examined by flow cytometry. Trypan blue was utilized to quench the fluorescence of nanoparticles adsorbed onto the exterior membrane of cells.

### Flow Cytometry Analysis of ROS Production in PMA-Stimulated RAW264.7 Macrophages

Typically, after 2 h of treatment of RAW264.7 macrophages with nanoparticles, cells were washed three times with PBS followed by incubation with 20 μM DHR123 in serum-free DMEM for 30 min. Then, RAW264.7 cells were washed with PBS and incubated with culture medium containing PMA at different concentrations for 1 h. After washing, RAW264.7 macrophages were harvested and analyzed by a FACS Canto II flow cytometer.

### Quantitative Analysis

RAW264.7 cells at 3 × 10^4^ per well were seeded in 96-well plates for semi-quantitative assays. After incubation with 50 μL of 100 μg/mL of a nanoparticle suspension in serum-free DMEM for 2 h, nanoparticle-treated cells were treated with 50 μL of serum-free DMEM containing different concentrations of PMA, and 20 μM DHR123 for an additional 1 h at 37 °C. At the same time, external standards of H_2_O_2_ mixed with 50 μg/mL HRP@HSNs were used to develop a calibration curve by plotting the fluorescence intensity versus the concentration of H_2_O_2_. The fluorescence intensity was measured with a microplate reader (BioTek Synergy Hybrid Reader) with excitation at 488 nm and emission at 530 nm. Using the established calibration curve, amounts of H_2_O_2_ in RAW264.7 cells stimulated with various amounts of PMA were calculated.

### Characterization

Transmission electron microscopic (TEM) images were taken on a JEOL JEM-1200 EX II operating at 100 kV. Images were recorded with a GatanOrius CCD camera. Samples were dispersed in 95% ethanol and dropped onto a carbon-coated copper grid, and then air-dried and examined. To verify HRP in the hollow spheres, a negative staining sample was stirred in 1% aqueous uranyl acetate (UA) for 1 h, and then centrifuged to remove the remaining UA. Finally, the sample was dispersed in ethanol and dropped onto the copper grid for imaging. Dynamic light scattering (DLS) and zeta potential measurements were performed on a Zetasizer Nano ZS (Malvern, UK). Optical images of RAW264.7 cells were obtained with a Zeiss Axio Observer Z1 inverted microscope.

## Results and Discussion

### Design and Synthesis of HSNs and HRP@HSNs

**Scheme 1:**
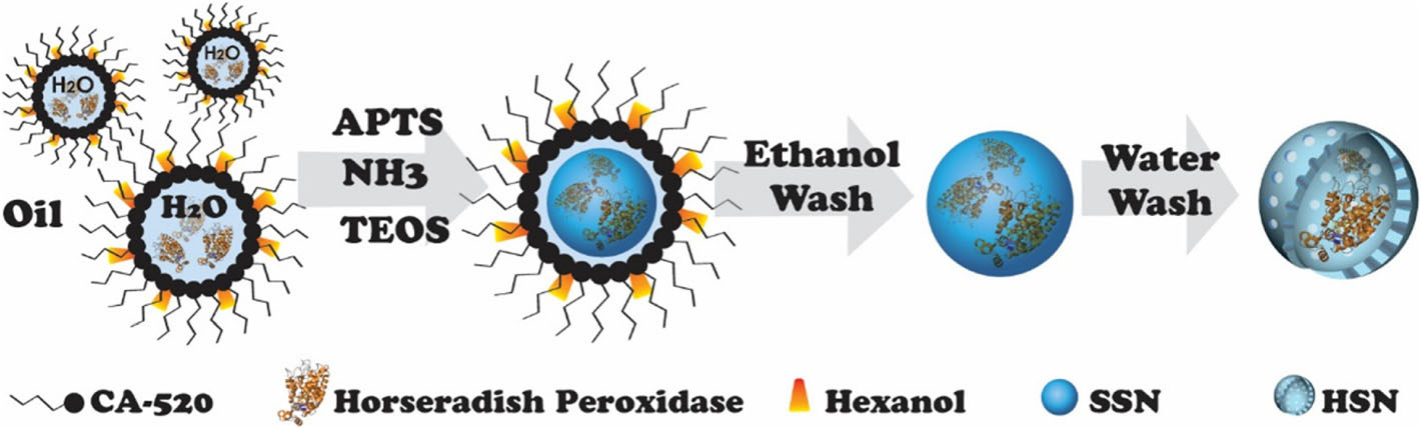
Flow chart of the synthesis of horseradish peroxidase-encapsulated hollow silica nanospheres (HRP@HSNs). APTMS, 3-aminopropyltrimethoxysilane; TEOS, tetraethyl orthosilicate; SSN, solid silica nanoparticle

**Fig. 1:**
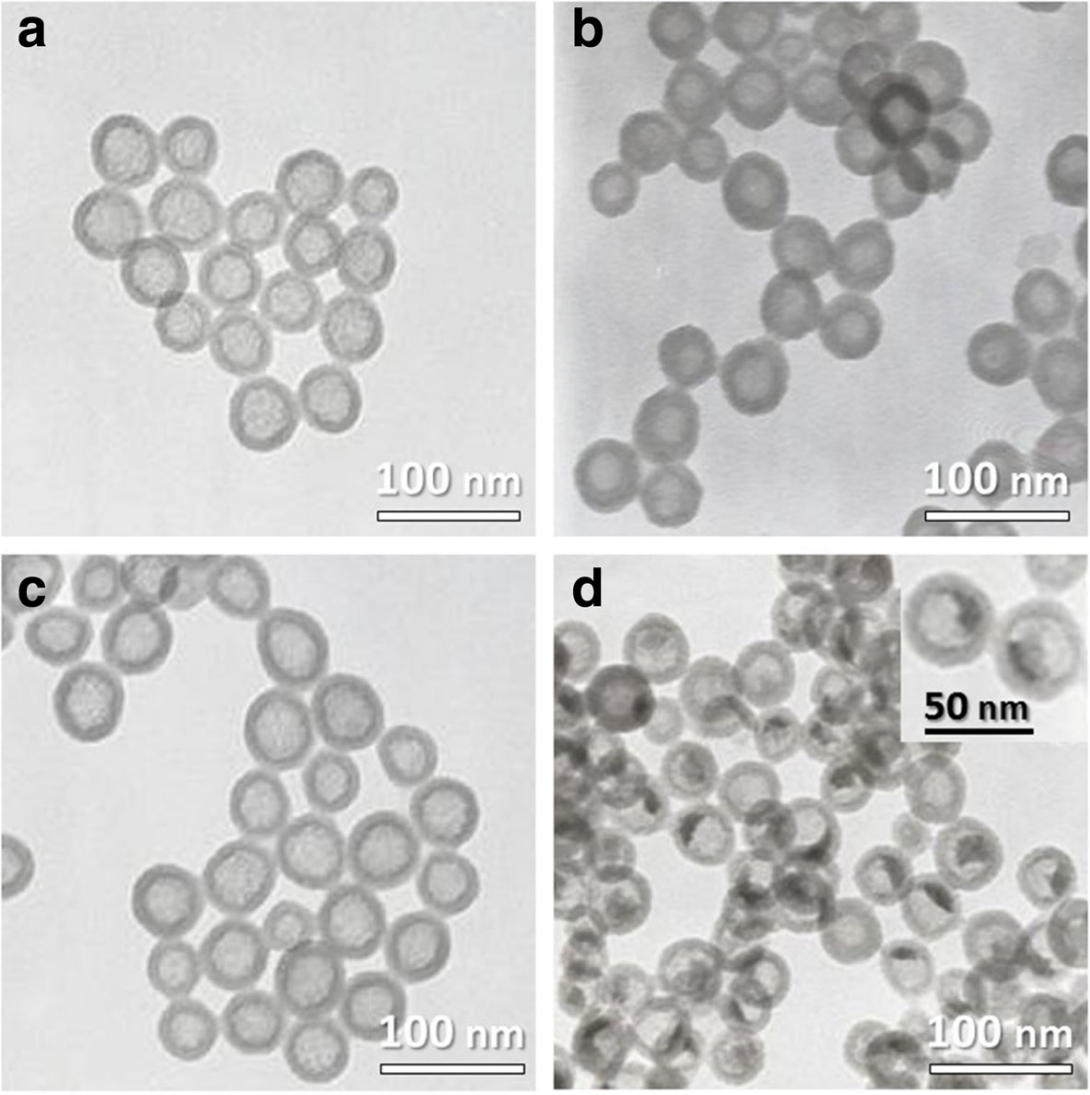
TEM images of **a** hollow silica nanospheres (HSNs), **b** HSNs stained with uranyl acetate, **c** horseradish peroxidase-encapsulated HSNs (HRP@HSNs), and **d** HRP@HSNs stained with uranyl acetate. Inset: an enlarged view

Typically, HSNs and HRP@HSNs were synthesized via an ammonia-catalyzed sol-gel process combined with a water-in-oil (w/o) microemulsion system according to our previous method [27, 28]. Scheme 1 illustrates the synthesis of HRP@HSNs. According to TEM images (Fig. 1), HSNs with and without encapsulated HRP showed an average diameter of 45 nm (Additional file 1: Figure S1). UA staining clearly displayed an enhanced electron density inside the HRP@HSNs, but no staining was observed outside the HRP@HSNs (Fig. 1b), indicating that the HRP enzymes were successfully entrapped within the interior cavity of HRP@HSNs.

The DLS measurements and zeta potential analyses performed at room temperature are shown in Table 1. DLS data showed that both HSNs and HRP@HSNs gave positive zeta potentials in water (pH ~ 6.5) with hydrodynamic diameters of 188 ± 4 and 184 ± 6 nm in water, respectively. However, when the nanoparticles were dispersed in serum-free DMEM, the hydrodynamic diameters increased to 1767 ± 94 nm for HSNs and 1598 ± 127 nm for HRP@HSNs. These indicate a small degree of aggregation of HSNs, but they were still well suspended in media. Meanwhile, the negative zeta potentials of both nanoparticles measured in media implied that some of ions and biomolecules from the biological milieu may have been adsorbed onto the nanoparticle surfaces [30, 31]. Under this condition, the positively charged surfaces of nanoparticles were covered by negatively charged substances, which rapidly caused aggregation of the nanoparticles through electrostatic interactions. To reduce non-specific aggregation and promote the colloidal stability of nanoparticles, bovine serum albumin (BSA) was introduced to the biological media [28]. Subsequently, the hydrodynamic diameters of HSNs and HRP@HSNs showed considerable decreased hydrodynamic diameters to 197 ± 43 and 195 ± 19 nm respectively.

**Table 1:** Dynamic light scattering (DLS) and zeta potentials of nanoparticles in water, Dulbecco’s modified Eagle’s medium (DMEM), and DMEM + bovine serum albumin (BSA)

Sample^a^	HSNs			HRP@HSNs		
Solution	H_2_O	DMEM	DMEM + BSA	H_2_O	DMEM	DMEM + BSA
DLS (d.nm) (±SD)	188 (± 4)	1767 (± 94)	197 (± 43)	184 (± 6)	1598 (± 127)	195 (± 19)
Zeta (mV) (±SD)	27.8 (± 0.4)	− 13.6 (± 0.7)	− 11.0 (± 0.9)	23.0 (± 1.2)	− 13.4 (± 0.3)	− 11.9 (± 0.4)

*HSN* hollow silica nanospheres, *HRP@HSNs* horseradish peroxidase-encapsulated HSNs

^a^All particles were measured at a concentration of 0.3 mg/mL. Each measurement was repeated at least three times

### HRP Entrapment Efficiency and Loading Capacity of HRP@HSNs

To investigate the efficiency and loading capacity of HRP entrapment, fluorescent dye (RITC)-labeled HRP was prepared (designated RITC-HRP). The fluorescence intensity of the RITC-HRP@HSNs was measured by suspending the nanoparticles in 1 M NaOH, and the amount of encapsulated RITC-HRP was determined according to a calibration curve established by plotting the fluorescence intensity versus the concentration of native RITC-HRP under the same conditions (Additional file 1: Figure S2). To study the effects of the enzyme concentration on the entrapment efficiency and loading capacity, three different amounts of HRP (11.1, 22.2, and 33.3 nmol) were introduced to the synthesis. It is worth noting that in this range of concentration, regardless of how much enzyme was introduced, the entrapment efficiency of enzymes for each of the three cases was about 6%. This low efficiency may be due to the fact that only a fraction of the microemulsion droplets nucleated and grew to HSN; most of the microemulsion droplets were not nucleated and remained in its small size of ~ 8 nm [25]. Future work may be needed for increasing the loading efficiency. However, the HRP loading capacity of HRP@HSNs gradually increased to 12.5 ± 1.2 μg HRP/mg HSNs when 33.3 nmol of HRP was used (Additional file 1: Table S1). This indicates that the HRP loading capacity can be controlled by the amount of enzyme present in the reaction.

### Cytotoxicity and Cellular Uptake of HSNs and HRP@HSNs

To evaluate the in vitro cytotoxicity of HSNs and HRP@HSNs, cell viability was examined by WST-1 assays. As shown in Additional file 1: Figure S3, no significant change in RAW264.7 cell proliferation was observed after treatments of nanoparticles for either 2 h, or 2 h followed by an additional 24 h of culture. No obvious effect on the cellular mitochondrial function caused by the silica nanoparticles was found at the indicated time points, regardless of the presence or absence of HRP inside HSNs.

Next, FITC-conjugated HSNs and HRP@HSNs were respectively prepared to investigate the concentration effect of nanoparticles on RAW264.7 labeling. Flow cytometric results (Additional file 1: Figure S4) showed that RAW264.7 cells were successfully labeled with FITC-HSNs and HRP@FITC-HSNs at different concentrations for 2 h in serum-free media. In both cases, dose-dependent increases in labeling efficiency were found, and more than 80% of RAW264.7 cells were labeled by exposure to nanoparticles at a concentration of > 50 μg/mL for 2 h. Properties such as high-efficiency intracellular labeling with a short incubation time, a relatively low dose of nanoparticles, and non-cytotoxicity make HRP@HSNs suitable for intracellular detection of ROS.

### Reactivity of HRP@HSNs to Various ROS

According to HRP enzyme activity assay using TMB as substrate, about 40% of the initial enzyme activity remained upon subsequent encapsulation of HRP into HSNs. This decrease in the observed specific activity of the encapsulated enzyme (moles of substrate converted per unit enzyme per unit time) could have resulted from mass transfer limitations, that occur when substrates cross the silica shell toward the HRP [32]. Nevertheless, the encapsulation strategy provides additional features, for example, the porous silica shell can protect HRP against proteolysis while allowing transport of small molecules of reactants and products [26, 27]. Taken together, the observed reactivity of HRP@HSNs to ROS, evaluated by incorporating a fluorescent probe (DHR123), could be resulted from a combination of the affinity of the nanoparticles as well as intrinsic property of HRP for ROS.

**Fig. 2:**
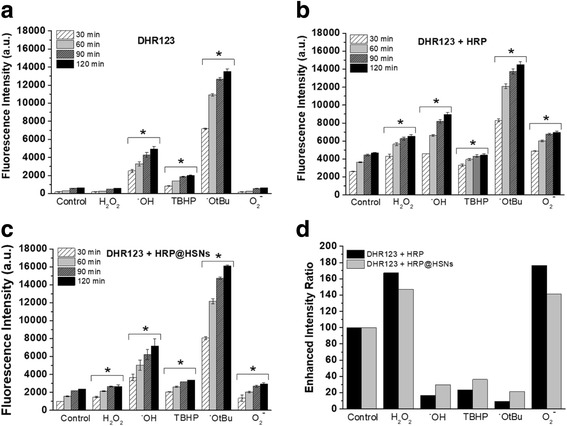
**a**–**c** Time-dependent fluorescence intensity of the reaction of selected reactive oxygen species (ROS) with **a** dihydrorhodamine 123 (DHR123), **b** DHR123 + horseradish peroxidase (HRP), and **c** DHR123 + horseradish peroxidase-encapsulated HSNs (HRP@HSNs). **d** Enhanced intensity ratio of the reaction of selected ROS with DHR123 + HRP and DHR123 + HRP@HSNs at 1 h. Data shown are for 20 μM of DHR123, 400 ng/mL of HRP, 50 μg/mL of HRP@HSNs, and 100 μM of ROS. (*p < 0.05 versus the control group at corresponding time points)

Cell-free systems were used to generate a variety of biologically relevant ROS, including hydrogen peroxide (H_2_O_2_), TBHP, hydroxyl radicals (•OH), tert-butoxy radicals (•OtBu), and superoxide (O_2_•^−^). First, DHR123 was incubated with a panel of ROS in the absence and presence of HRP or HRP@HSNs followed by measuring the fluorescence intensity of the product rhodamine 123 (R123). As shown in Fig. 2, regardless of which type of ROS was employed, the fluorescence intensity was measured in a time-dependent manner (30, 60, 90, and 120 min). However, apparent differences in intensity among various ROS depend on the intrinsic properties of DHR123. On the one hand, in agreement with a previous study [33], Fig. 2a shows that neither H_2_O_2_ nor O_2_•^−^ could oxidize DHR123 to R123. Additionally, DHR123 exhibited higher reactivity for •OtBu and •OH radicals over other ROS. With the catalytic activity of HRP, remarkable increases in the fluorescence intensity were observed in the presence of native HRP and HRP@HSNs as shown in Fig. 2b, c. It was noted that the higher fluorescence intensity found in the case of native HRP compared to HRP@HSNs at the same reaction time was positively correlated with their observed enzyme activities.

To allow a direct comparison between various ROS, data at a time interval of 60 min were selected and reported as relative fluorescence intensity normalized to the control (Additional file 1: Figure S5). Subsequent analysis of the enhanced intensity ratio was shown by dividing the relative fluorescence intensity of DHR123 + HRP or DHR123 + HRP@HSNs by DHR123 (Fig. 2d). In both HRP-containing cases, a similar trend of the enhanced intensity ratio over various ROS as well as significant increases in the reactivity of DHR123 to H_2_O_2_ and O_2_•^−^ were observed, demonstrating that the encapsulated HRP gave a high degree of intrinsic enzyme activity, and the silica shells of HRP@HSNs allowed transport of small molecules to carry out selective bio-catalysis.

### Intracellular ROS Detection with HRP@HSNs

**Fig. 3:**
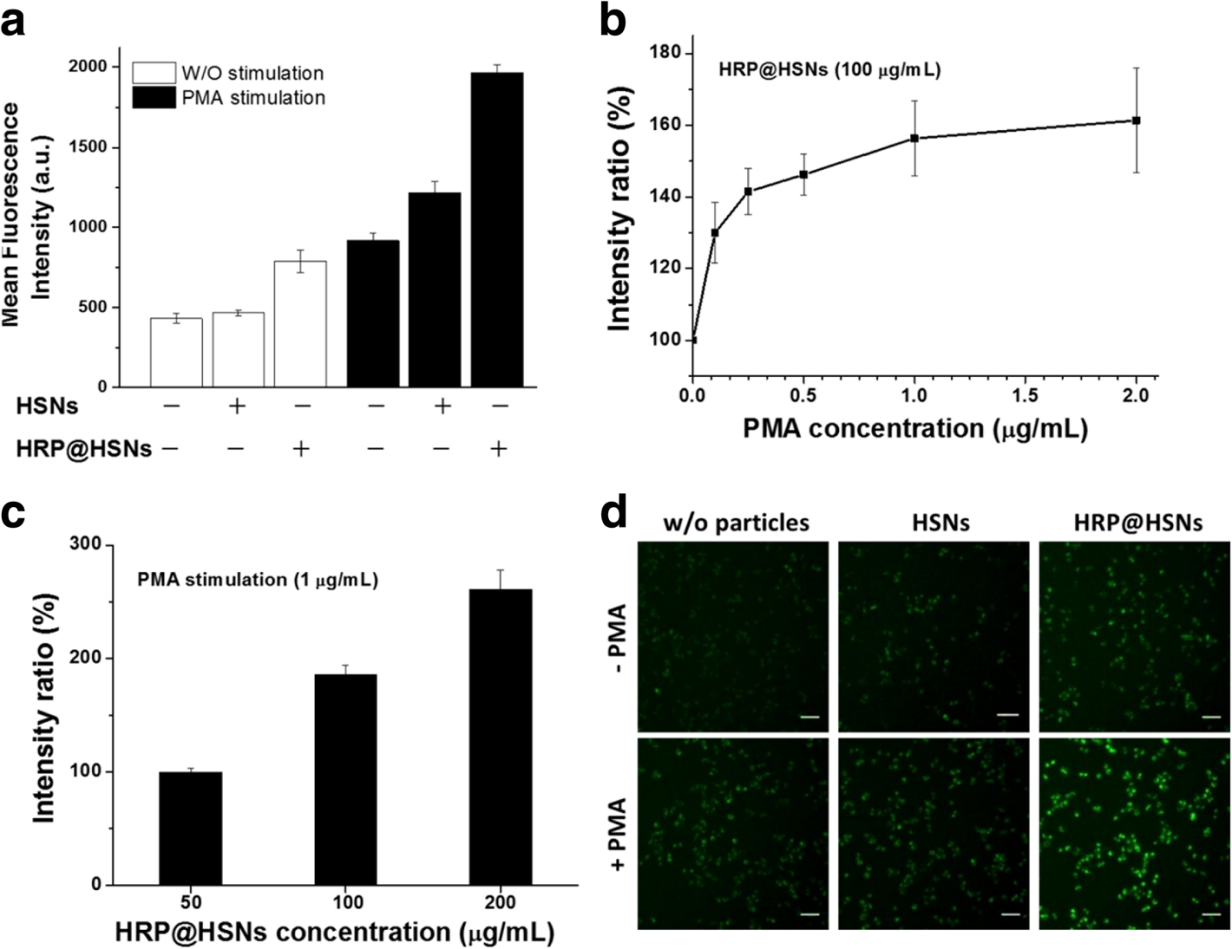
**a** Flow cytometry analyses of RAW264.7 macrophages stimulated with and without phorbol 12-myristate 13-acetate (PMA) in the presence and absence of nanoparticles. **b** PMA and **c** horseradish peroxidase-encapsulated HSNs (HRP@HSNs) concentration dependently altered the fluorescence of RAW264.7 macrophages. **d** The representative fluorescence images of RAW264.7 macrophages at the indicated conditions. Scale bars 50 μm

To assess the ROS detection functionality of HRP@HSNs inside cells, RAW264.7 macrophages were incubated with HRP@HSNs for 2 h followed by washing and then incubating with DHR123 (20 μM) for 30 min. Subsequently, cells were washed and treated with PMA (1 μg/mL) for an additional 1 h. It is known that stimulating macrophages with PMA results in production of superoxide, which is dismuted to hydrogen peroxide by superoxide dismutase or by spontaneous dismutation [34–36]. Thus, PMA can function as a stimulant to generate H_2_O_2_ in RAW264.7 macrophages to evaluate the intracellular H_2_O_2_-sensing capability of HRP@HSNs. As shown in Fig. 3a, both cases of RAW264.7 macrophages cultured alone and cultured with HSNs showed weak fluorescence in the flow cytometric analysis, indicating that non-stimulated cells produced a weak basal level of ROS, where no significant ROS were induced in the presence of HSNs. In addition, cells treated with HRP@HSNs showed significant intensity increase (Fig. 3a), suggesting that the delivered HRP@HSNs gave extra catalytic activity inside cells.

For stimulation experiments, PMA-treated cells typically generated more than twofold higher levels of R123 fluorescence compared to unstimulated cells. In addition, cells treated with HRP@HSNs had the highest level of fluorescence, followed by HSNs and then cells alone. It was noted that treating the stimulated RAW264.7 macrophages with HSNs resulted in a small increase in fluorescence intensity compared to that of the control. This result suggested that cellular stress responses are triggered extremely rapidly, and sensitive to external stimuli, including exposure to nanoparticles [37]. In addition, both PMA (0.1, 0.25, 0.5, 1, and 2 μg/mL) and HRP@HSNs (50, 100, and 200 μg/mL) induced expression of R123 in a dose-dependent manner, as evident in Fig. 3b, c.

**Fig. 4:**
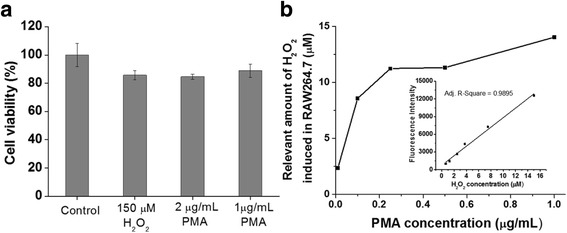
**a** WST-1 assay of RAW 264.7 macrophages after treatments of exogenous H_2_O_2_ or stimulation with phorbol 12-myristate 13-acetate (PMA) for 1 h. **b** Detection of the concentration of H_2_O_2_ endogenously produced by RAW264.7 macrophages under various concentrations of the PMA stimulant in the presence of horseradish peroxidase-encapsulated hollow silica nanospheres (HRP@HSNs) and dihydrorhodamine 123 (DHR123). Inset: a calibration curve obtained from the external standards of H_2_O_2_ mixed with HRP@HSNs and DHR123

In accordance with the flow cytometric analysis, Fig. 3d displays the representative fluorescence images of RAW264.7 macrophages stimulated with and without PMA in the presence and absence of nanoparticles. The system was capable of visualizing endogenous H_2_O_2_ generation in RAW264.7 cells, and the weakest fluorescence intensity was observed in cells treated with HRP@HSNs followed by PMA stimulation. As shown in Fig. 4a, the cell viability of RAW264.7 macrophages in the presence of the stimulant PMA or exogenous H_2_O_2_ was examined by WST-1 assays. Whereas ROS have been implicated in apoptosis [38], only a small effect on cell viability was found at the indicated time point, making the following semi-quantitative analysis practical and meaningful.

### Application of HRP@HSNs In Vitro for Quantitative Analysis of H_2_O_2_

To evaluate the capacity of HRP@HSNs for quantifying endogenous hydrogen peroxide produced in PMA-stimulated RAW264.7 cells, a calibration curve from the exogenous H_2_O_2_ experiment, with a detection range of 0.625~15 μM, was established by microplate measurements (Fig. 4b, inset). The standard calibration curve appears to be linear as expected. Then, RAW264.7 cells were treated with 100 μg/mL of HRP@HSNs for 2 h, followed by co-incubation with various concentrations of PMA and 20 μM of DHR123 at 37 °C for 1 h. After that, the concentration of H_2_O_2_ endogenously produced by PMA-stimulated RAW264.7 cells was determined by measuring the fluorescence intensity, followed by conversion using the established calibration curve. Notably, because most of the HRP@HSNs were uptaken within the cells, the H_2_O_2_-triggered fluorescence of R123 could be attributed to intracellular enzyme-catalyzed reactions rather than the extracellular contribution. Although H_2_O_2_ is able to diffuse across biomembranes, due to its limited diffusion and rapid enzymatic consumption inside cells, concentration gradients of H_2_O_2_ are formed across membranes [39, 40]. Typically, under normal physiological conditions, H_2_O_2_ has an extracellular concentration estimated at 10^− 7^~10^− 6^ M, which is about 10-fold higher than that observed in intracellular fluid [1, 41, 42]. In pathological conditions, extracellular concentrations of H_2_O_2_ are in the range of 10~50 μM and are additionally elevated to as high as 10^− 4^ M in apoptosis [1]. As shown in Fig. 4b and Additional file 1: Table S2, endogenous hydrogen peroxide caused by PMA-stimulated RAW264.7 cells was created in a dose-dependent manner and produced at levels of about 10 μM when the concentration of PMA used exceeded 0.25 μg/mL. Taken together, these results indicate that HRP@HSNs were capable of detecting semi-quantitatively endogenous the concentration of hydrogen peroxide of RAW264.7 macrophages under oxidative stress conditions.

## Conclusions

In summary, we have demonstrated that hollow silica nanospheres encapsulating HRP can be synthesized via a microemulsion-templating system and act as intracellular fluorescent ROS sensors. The shells of HRP@HSNs are permeable to small molecules, such as the enzyme substrates, which allows them to react with large enzyme payloads in the hollow cavity. Both the effective intracellular delivery and satisfactory catalytic activity of HRP@HSNs significantly enhance reduction-triggered fluorescence and constitute the ability of semi-quantitative measurements of endogenous H_2_O_2_ in RAW264.7 macrophages under oxidative stress conditions.

Because the concentration and location of H_2_O_2_ in eukaryotic cells strongly rely on the types of cells, and cellular compartments [1], specific targeting of tumor cells or organelles could further be achieved by surface modification of HRP@HSNs with monoclonal antibodies or peptides. Also, non-enzymatic H_2_O_2_ detection could be realized by replacing the interior nanoreactors of HRP with nanoparticles [43, 44] or boronate-based fluorescent probes [42, 45]. Future efforts should be devoted to maximizing the sensitivity and specificity for H_2_O_2_ as well as enabling more informative designs of next-generation nanomaterials. Such hollow capsules could be a promising platform for modern nanomedicines that aims to simultaneously image, sensing, and deliver therapeutic molecules specifically to defective cells.

## Additional file

Additional file 1: Size distribution histograms of HSNs and HRP@HSNs. Calibration curve of fluorescence intensity versus RITC-HRP. Flow cytometry, cytotoxicity, and cell proliferation assays. Entrapment efficiency and loading capacity of HRP@HSNs. (DOC 450 kb)

## Abbreviations

APTMS: 3-Aminopropyltrimethoxysilane
BSA: Bovine serum albumin
DHR123: Dihydrorhodamine 123
DLS: Dynamic light scattering
FITC: Fluorescein isothiocyanate
HRP: Horseradish peroxidase
HSNs: Hollow silica nanospheres
Igepal CA-520: Polyoxyethylene (5) isooctylphenyl ether
mETC: Mitochondrial electron transport chain
PET: Positron emission tomography
PMA: Phorbol 12-myristate 13-acetate
R123: Rhodamine 123
RITC: Rhodamine B isothiocyanate
ROS: Reactive oxygen species
SSN: Solid silica nanoparticles
TBHP: Tert-butyl hydroperoxide
TEM: Transmission electron microscopic
TEOS: Tetraethyl orthosilicate
TMB: 3,3′5,5′-Tetramethylbenzidine
