# Supplementary material for: Horseradish Peroxidase-Encapsulated Hollow Silica Nanospheres for Intracellular Sensing of Reactive Oxygen Species
Source: Nanoscale Res Lett. 2018 Apr 24;13:123. doi: 10.1186/s11671-018-2527-0 (PMC5915989; doi:10.1186/s11671-018-2527-0)

**Additional file 1**

**Horseradish Peroxidase-encapsulated Hollow Silica Nanospheres for Intracellular Sensing of Reactive Oxygen Species**

Hsin-Yi Chen^a^, Si-Han Wu*^bc^, Chien-Tsu Chen^d^, Yi-Ping Chen^bc^, Feng-Peng Chang^a^, Fan-Ching Chien^e^ and Chung-Yuan Mou*^a^

^a^ Department of Chemistry, National Taiwan University, Taipei 10617, Taiwan ^b^Graduate Institute of Nanomedicine and Medical Engineering, College of Biomedical Engineering, Taipei Medical University, Taipei 11031, Taiwan ^c^ International PhD Program in Biomedical Engineering, College of Biomedical Engineering, Taipei Medical University, Taipei 11031, Taiwan ^d^Department of Biochemistry, Taipei Medical University, Taipei 11031, Taiwan ^e^Department of Optics and Photonics, National Central University, Taoyuan County 32001, Taiwan *To whom correspondence should be addressed.

E-mail: smilehanwu@tmu.edu.tw; cymou@ntu.edu.tw; Fax: +886-2-23660954; Tel: +886-2-33665232


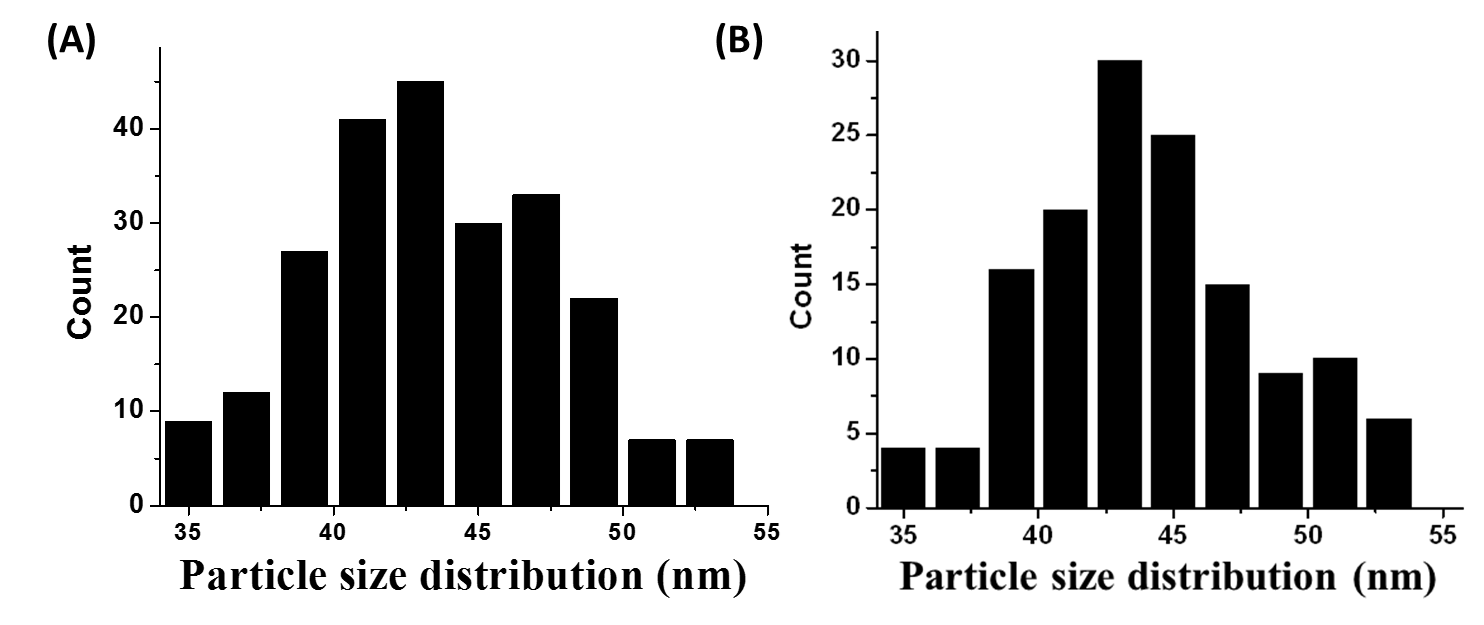
**Figure S1.** Size distribution histogram of (A) hollow silica nanospheres (HSNs); (B) horseradish peroxidase-encapsulated hollow silica nanospheres (HRP@HSNs) calculated from TEM images.

**
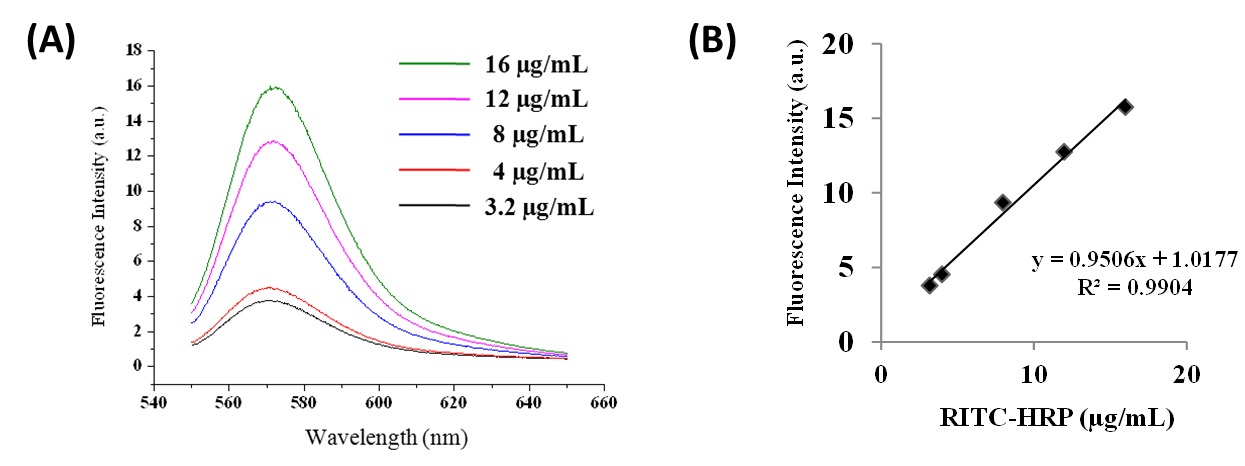
**

**Figure S2.** Calibration curve of the fluorescence intensity versus rhodamine B isothiocyanate (RITC)-horseradish peroxidase (HRP). (A) Fluorescence spectra of different concentrations of RITC-HRP in 1M NaOH. (B) The plot of the fluorescence intensity versus the concentration of RITC-HRP.


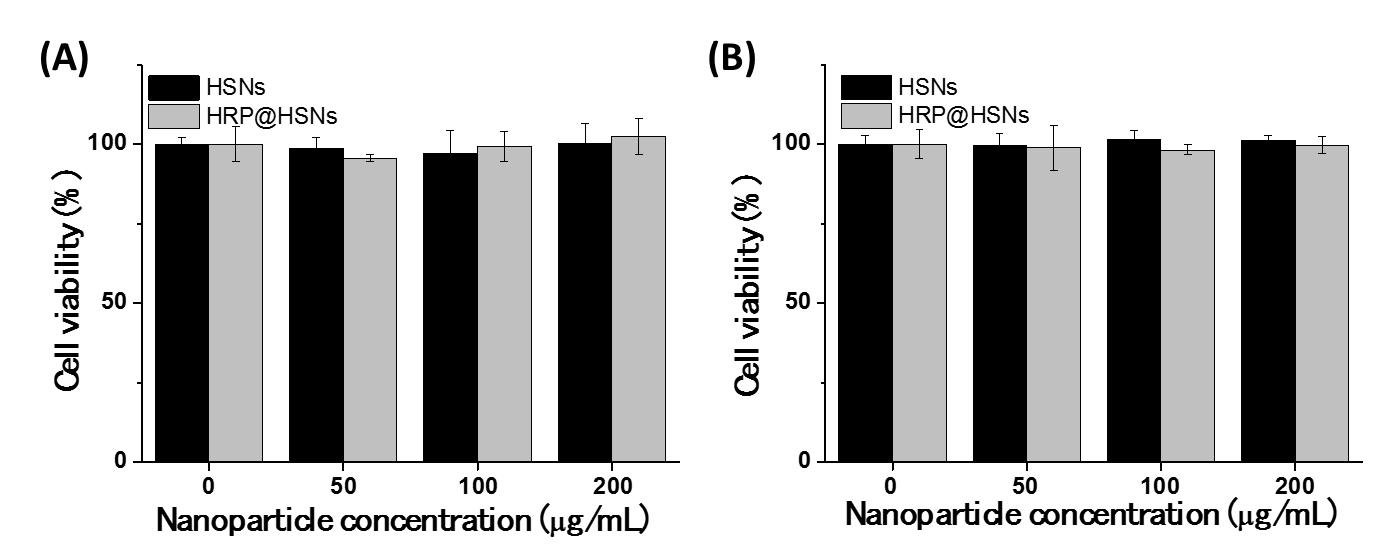


**Figure S3.** (A) Cytotoxicity and (B) cell proliferation of RAW264.7 macrophages treated with hollow silica nanospheres (HSNs) and horseradish peroxidase-encapsulated HSNs (HRP@HSNs). Cells were respectively incubated with HSNs and HRP@HSNs at different doses (0, 50, 100, and 200 µg/mL) for 2 h. The WST-1 assay was conducted (A) after 2 h nanoparticle treatment or (B) after 2 h nanoparticle treatment followed by cell growth for 24 h.


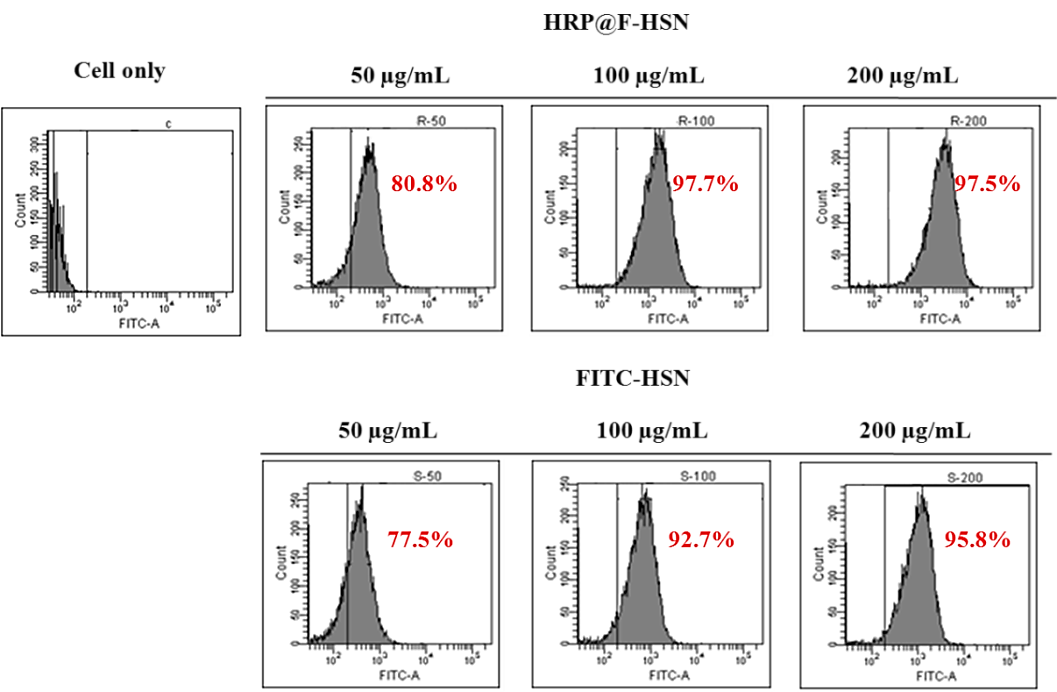


**Figure S4.** Efficiency of cellular uptake of horseradish peroxidase-encapsulated FITC hollow silica nanospheres (HRP@FITC-HSNs) and FITC-HSNs (50, 100, and 200 µg/mL) by RAW 264.7 cells for 2 h.


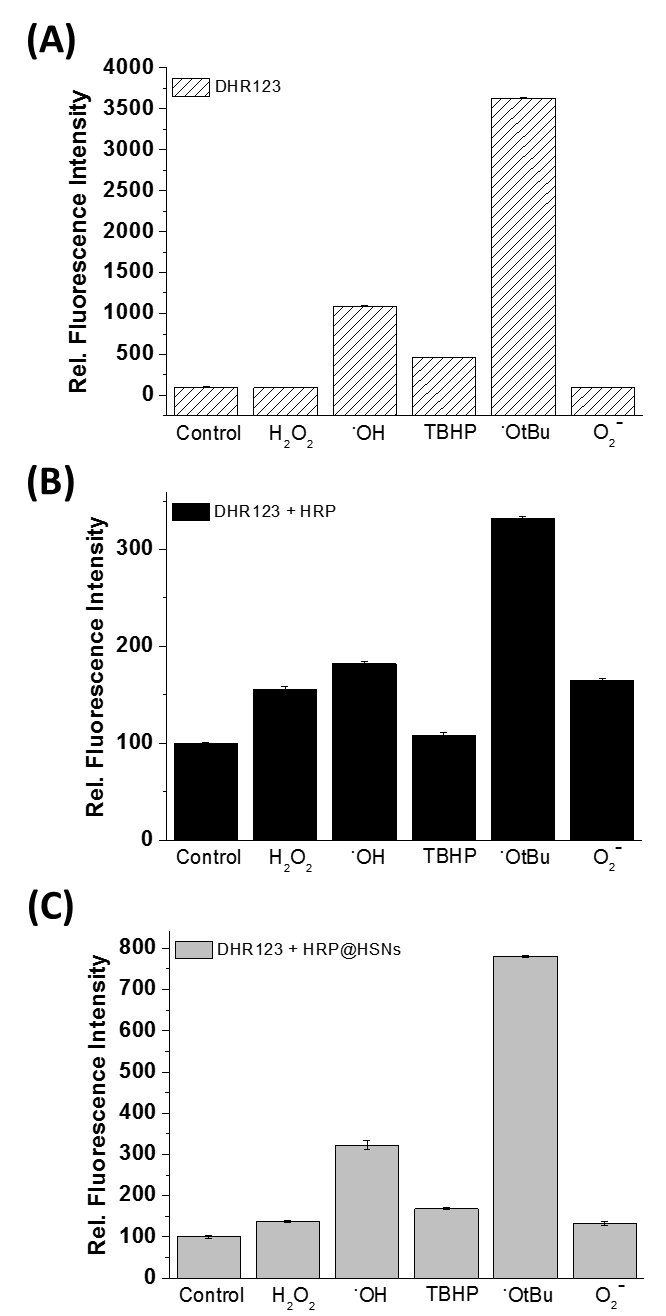


**Figure S5.** The relative fluorescence intensity of the reaction of selected reactive oxygen species (ROS) with (A) dihydrorhodamine 123 (DHR123), (B) DHR123+horseradish peroxidase (HRP) and (C) DHR123+HRP-encapsulated hollow silica nanospheres (HRP@HSNs) at a time interval of 60 min. Signals were calculated by dividing the intensity of the experimental condition by the intensity of control.

**Table S1.** Entrapment efficiency and loading capacity of horseradish peroxidase-encapsulated hollow silica nanospheres (HRP@HSNs).

**
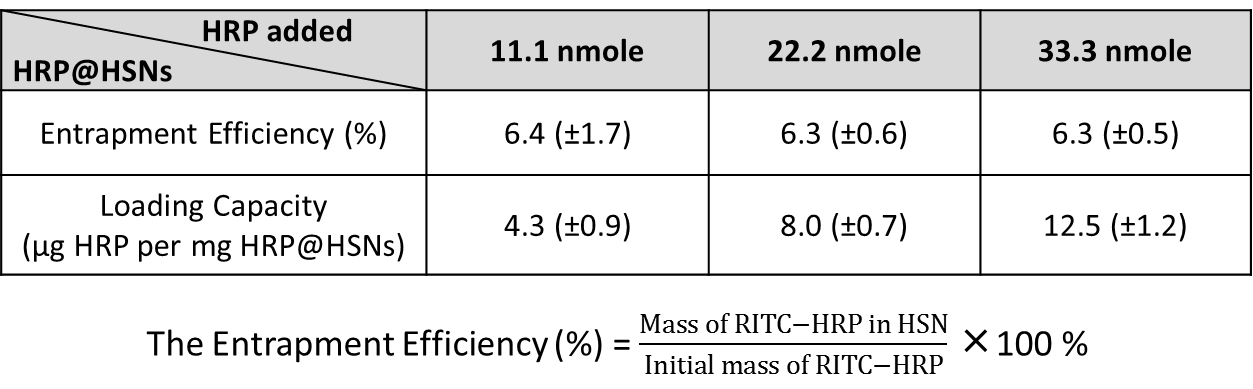
**

**Table S2.** Concentration of hydrogen peroxide endogenously caused by phorbol 12-myristate 13-acetate (PMA)-stimulated RAW264.7 cells.


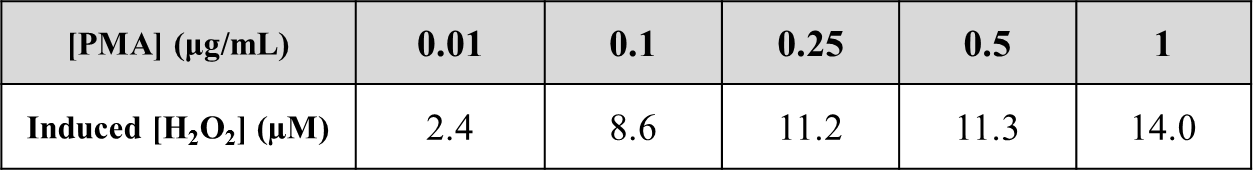

Supplement: Supplementary file 1 — Size distribution histograms of HSNs and HRP@HSNs. Calibration curve of fluorescence intensity versus RITC-HRP. Flow cytometry, cytotoxicity, and cell proliferation assays. Entrapment efficiency and loading capacity of HRP@HSNs. (DOC 450 kb) [file 11671_2018_2527_MOESM1_ESM.doc]
